# Does public reporting influence antibiotic and injection prescribing to all patients? A cluster-randomized matched-pair trial in china

**DOI:** 10.1097/MD.0000000000003965

**Published:** 2016-07-01

**Authors:** Chenxi Liu, Xinping Zhang, Xuan Wang, Xiaopeng Zhang, Jie Wan, Fangying Zhong

**Affiliations:** School of Medicine and Health Management, Tongji Medical School, Huazhong University of Science and Technology, Wuhan, Hubei Province, China.

**Keywords:** a matched-pair randomized trial, antibiotics, difference-in-difference, injections, public reporting, rational use of medicine

## Abstract

The inappropriate use and overuse of antibiotics and injections are serious threats to global population, and the public reporting of health care performance (PRHCP) has been an important instrument for improving the quality of care. However, existing evidence shows a mixed effect of PRHCP. This study is to explore the potential effectiveness of PRHCP that contributes to the convincing evidence of health policy and reform.

This study was undertaken in Qian Jiang City, applying a matched-pair cluster-randomized trial. Twenty primary care institutions were treated as clusters and were matched into 10 pairs. Clusters in each pair were randomly assigned into a control or an intervention group. Physicians’ prescribing information was publicly reported to patients and physicians monthly in the intervention group from October 2013. A total of 748,632 outpatient prescriptions were included for difference-in-difference (DID) regression model and subgroups (SGs) analysis.

Overall, PRHCP intervention led to a slight reduction in the use of combined antibiotics (odds ratio [OR] = 0.870, *P* < 0.001) and slowed the average expenditure increase of patients (coefficient = −0.051, *P* < 0.001). SG analysis showed the effect of PRHCP varied among patients with different characteristics. PRHCP decreased the probability of prescriptions requiring antibiotics, combined antibiotics, and injections of patients aged 18 to 64 years old (OR < 1), and all results were statistically significant. By contrast, the results of elderly and minor patients with health insurance showed that PRHCP increased their probability of prescriptions requiring antibiotics and injections. PRHCP slowed the increase of average expenditure of most SGs.

PRHCP intervention can influence the prescribing pattern of physicians. Patient factors such as age and health insurance influence the effect of PRHCP intervention, which imply that PRHCP should be designed for different patients. Patient education, aiming at radically changing attitudes toward antibiotics and injections, should be taken to promote the effectiveness of public reporting in China.

## Abstract 2

Supplemental Digital Content is available in the text

## 1 Introduction

The rational use of medicine is an important aspect of quality of care,^[1]^ and irrational use of antibiotics and injections have become an outstanding problem all over the world, especially in low- and middle-income countries.^[2,3]^ It has been reported that over 50% patients were prescribed with antibiotics or injections in China,^[2]^ which are double compared with the appropriate rate.^[2,4]^ Irrational use of antibiotics results in drug adverse reactions and antimicrobial resistance, threatening the global population. It also increases medical cost, extend hospital stay, and lead to patient death^[5–9]^; on the other hand, overuse of injections can increase patient's risk infected by viruses, such as hepatitis C and AIDS.^[10]^ Irrational use of antibiotics and injections has caused increasing concerns and actions, aiming at addressing the issue, have been taken all over the world.^[11]^

Public reporting of health care performance (PRHCP) is becoming an important quality improvement instrument in most developed countries^[12,13]^ and has proliferated over the past decades.^[14]^ The United States and the United Kingdom have led the modern public disclosure movement of quality of care. This popular instrument for improving care quality has been proposed as a mechanism^[15,16]^ for providing transparency and increasing the motivation of health care providers.^[14,17]^

However, existing evidence on the efficacy of PRHCP is mixed. Several studies report that PRHCP stimulates quality improvement activity,^[18]^ but some studies disagree.^[19–22]^ Thus, the effect of PRHCP intervention is not clear.^[14,18]^

Many factors determine whether PRHCP will work as expected, and patient factor is an important aspect.^[23]^ Patient characteristics, such as age and insurance status, may assist or hinder efforts to improve quality.^[6,24–27]^ Another important issue is the design and implementation quality of the PRHCP intervention. A systematic review on PRHCP, which includes 45 articles from 1987 to 2008, showed that design and implementation, if they are sufficiently improved, may increase the effect of PRHCP intervention.^[18]^ Therefore, PRHCP must be designed and implemented appropriately, and it is also important to identify appropriate patients for intervention to make PRHCP effective.

The strength of the research designs may also contribute to the explanation of the mixed findings. Little evidence uses controlled experimental designs to evaluate the effect of PRHCP,^[14]^ and most studies use performance data before and after the release to evaluate the effect of PRHCP.^[12]^ In addition, the regression analysis of the difference-in-difference (DID) model is a powerful method to estimate the net effect of policy intervention.^[28]^ This method allows analysts to control the confounding influences of independent variables and makes causal claims about the effects of the intervention.^[1,3]^

China has conducted several public reporting movements to improve the quality of care,^[29]^ but scant evidence is available regarding the effect of PRHCP intervention.^[30]^ The 1st objective of the study is to explore the potential effectiveness of PRHCP intervention. The 2nd objective is to identify the appropriate patients who would benefit from PRHCP intervention, which contributes to convincing evidence for health policy and reform. Meanwhile, the design and implementation details of PRHCP intervention are also reported, which may contribute to the small but increasing practice and experience of PRHCP intervention.

## 2 Methods

### 2.1 Settings

Hubei Province is located in south central China and has a population of 60 million and a Gross Domestic Product (GDP) of 2225 billion (Yuan in 2012), which ranks this area in the middle range of all provinces. Qian Jiang City is a typical county in central Hubei Province. This study was undertaken in 20 primary care institutions in Qian Jiang City. Qian Jiang City was chosen purposely in consideration of its excellent hospital information system and good collaboration relationship with the local government that guaranteed that all the designed interventions could be implemented as planned.

There are average 27 physicians worked in each primary care institution in Qian Jiang City. And all the primary care institutions and physicians are selectable to all patients, there is no registration or assignment for patients that he must see specialized physicians. This study was approved by the Ethical Review Committee of Tongji Medical College, Huazhong University of Science and Technology (No. IORG0003571).

### 2.2 PRHCP experimental design

A randomized matched-pair trial design was applied in this study. Technique for Order Preference by Similarity to Ideal Solution is a multicriteria decision analysis method and was adopted to match all the 20 participating institutions. The procedure of this method is as following:

1. Generate a positive ideal alternative according the data provided.
2. Generate a negative ideal alternative according the data provided.
3. Calculate the geometric distance of each participating institution between positive and negative ideal alternatives.

Nine institutional characteristics were considered in Technique for Order Preference by Similarity to Ideal Solution, which were presented in Table 1. The participating institutions were ranked according to their geometric distances and adjacent institutions were paired. Institutions in each pair were randomly assigned into a control or an intervention group.

**Table 1:**
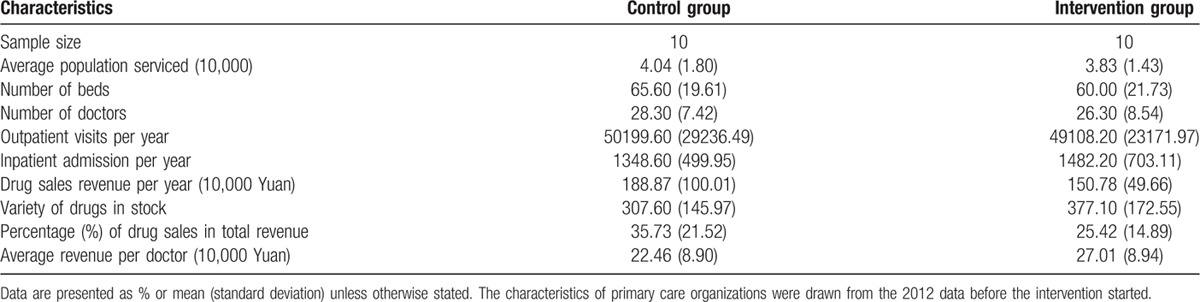
Characteristics of participating institutions.

The PRHCP intervention was implemented in the intervention group. All primary care institutions were exposed to the same context in Qian Jiang City, with the PRHCP intervention as the only exception.

### 2.3 PRHCP intervention design

The PRHCP intervention designed focused on the following important aspects.^[14,18,23,31,32]^

#### 2.3.1 Physician concerns about their reputation or market share

It is very important that physicians must care about the reporting information. Overuse of antibiotic and injection has been treated as a common form of inappropriate use of prescribing pharmaceuticals and has been treated as core dimensions to evaluate the quality of physicians’ prescribing by WHO. The inappropriate use of antibiotics and injection, as well as medical expenses, is the most prominent problem that is now being intensely regulated in China. Considering the prevalence and significance, public reporting of this information may be threat to physicians’ reputations or market share. Thus, the percentage of prescriptions requiring antibiotics or injections and the average expenditure of patients were selected.

#### 2.3.2 Truth of the reporting information

This aspect decided whether the public will use the reporting information and whether PRHCP will work as expected. To increase the trust of patients and physicians, we state the reporting information is calculated monthly using the data extracted from the electronic health information system. Moreover, a brief explanation about the calculation method is also given in the public reporting information.

#### 2.3.3 Easy to understand reporting information

Reporting information must be displayed in such a way that readers can effortlessly identify the better physician. Thus, a concise and explicit outcome chart and prescription ranking information are arranged appropriately for physicians because they are familiar with the relevant professional conceptions. We added a brief explanation, which includes the knowledge of antibiotics and injection overuse, for patients to have a better understanding.

#### 2.3.4 Public reporting indicators of PRHCP intervention

Three indicators, at both physician level and institutional level, were calculated for public reporting, including:

1. Antibiotic prescribing rate (%) = Number of antibiotic prescriptions/total number of a physician's (or an institution's) prescriptions in 1 month × 100%.
2. Injection prescribing rate (%) = Number of injection prescriptions/total number of a physician's (or an institution's) prescriptions in 1 month × 100%.
3. Average expenditure of patients (¥ Yuan) = Total expenditure of prescriptions/total number of a physician's (or an institution's) prescriptions in 1 month.

For example, if the antibiotic prescribing rate is 60% for physician Lee, it means that 60% of physician Lee's patients have been prescribed with antibiotics before. Antibiotic prescribing rate can indicate the probability of a physician or an institute giving patients antibiotic prescriptions. The specific performance in antibiotic prescribing for physician Lee in September 2013 was calculated based on the following formula: 
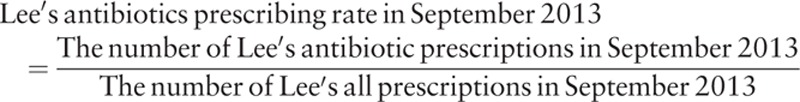


### 2.4 PRHCP intervention implementation

The PRHCP intervention was implemented in the intervention group, started on October 1, 2013 and ended on August 31, 2014. The content of public reporting information contained (For details of public reporting information, see Supplementary file, S1 file):

1. Three public reporting indicators: percentage of prescriptions requiring antibiotics, percentage of prescriptions requiring injections, and average expenditure of patients. All 3 indicators were calculated and ranked both at the physician and institutional levels.
2. A brief explanation about the knowledge of antibiotics and injection overuse.
3. A brief explanation about calculation method of the outcomes.

The league tables were printed in a 1.2 m × 0.8 m poster and displayed on a bulletin board in the lobby of outpatient departments during the period of intervention. Hard copies of public information poster were submitted to each physician involved in this study monthly in the intervention group.

The information was updated monthly and available on the 1st week of each month. A total of 14 investigators were recruited in postgraduate students for compliance assurance. They were trained prior to the start of intervention and randomly assigned to monitor the implementation of intervention measures every month. To avoid the investigator bias, the grouping of investigators was reassigned every 3 months. All the measures were implemented as designed.

Moreover, there was publicity of information for patients every 3 months; each of the publicity lasted for 3 days. Investigators introduced the poster to patients, instructed them to use it, and answered their questions about indicators or calculation methods.

### 2.5 Data collection and cleaning

The characteristics of the 20 institutions were investigated through their respective administrators before PRHCP intervention.

All prescription data for outpatients were collected, which covered a period of 13 months prior to the intervention (September 1, 2012 to September 31, 2013) and 11 months after the start of the intervention (October 1, 2013 to August 31, 2014). A total of 748,632 effective electronic prescriptions (Table 2) were cleaned for data analyses. Patient information was anonymized prior to analysis and the demographic information about outpatients, such as gender, age, and insurance status, as well as medical expenditure. Patient characteristics were grouped as follows: 2 groups based on gender – male and female; 3 groups based on age – juveniles (under 18 years old), adults (18–64 years old), and elderly (over 65 years old); and 2 groups based on payment status – with and without health insurance.

**Table 2:**
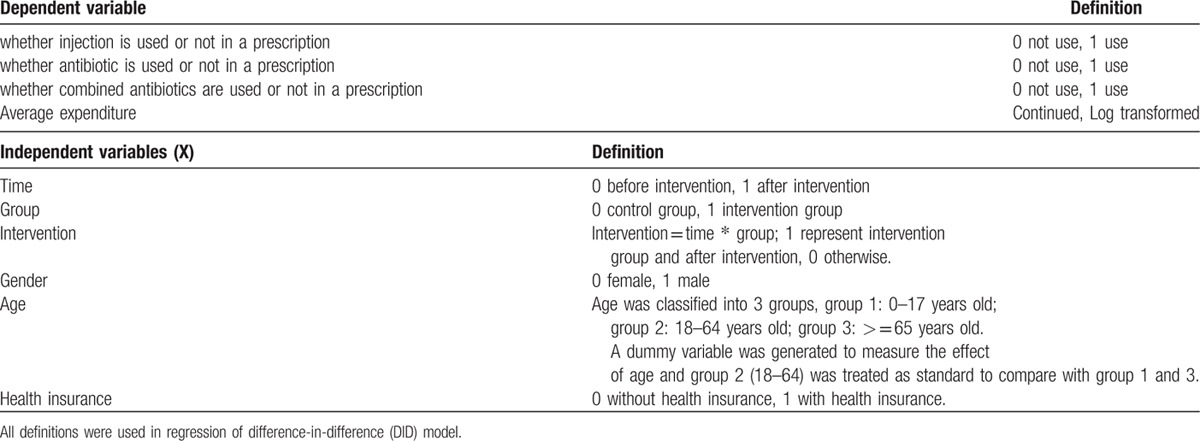
Definitions of independent and dependent variables.

If one institution or individual wants to request the data, please contact Health Bureau of Q City (http://www.qjsws.gov.cn/).

### 2.6 Statistic analysis

#### 2.6.1 Indicators used to evaluate PRHCP intervention

In this study, we examined the extent to which the PRHCP intervention changed the quality of care in relation to those indicators, such as:

1. Whether antibiotic is used or not in a patient prescription.
2. Whether combined antibiotics are used or not in a patient prescription.
3. Whether injection is used or not in a patient prescription.
4. Average expenditure of patients (including consultations, diagnostic tests, and prescriptions).

#### 2.6.2 Model of DID regression analysis

A regression analysis of the DID model was employed to evaluate the effect of the intervention.^[33,34]^

Logistic regression was used for binary dependent variables (indicators 1, 2, and 3). Whether antibiotics, injections, or combined antibiotics were used or not were generated according to the prescribing information of every patient. For each patient, the probability P of the occurrence of a dichotomous outcome Y can be described as: 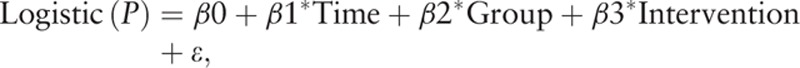


For the continuous dependent variable (indicator 4), a least squares regression model was used, which can be expressed as: 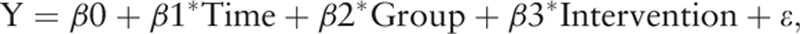


Considering that the average expenditure was skewed, the indicator was log transformed for the regression analysis.

#### 2.6.3 Basic interpretation of DID model

The coefficients β0 and β1 represent the constant term and effect of time, respectively. Moreover, β2 represents the difference between the control and intervention groups, and β3 is the net effect of intervention. Time effect and seasonality were included as adjustment factors. Robust standard error, based on the estimation of White Z test, was applied to adjust ordinary least squares regression model. Every single patient was considered as analytical unit, and to control the confounding effect, the gender, age, and health insurance of a patient were also included in this model.

To determine the kind of patient who would benefit from PRHCP intervention, 12 subgroups (SGs) were classified according to gender, male, and the ownership of health insurance. The results of the SGs analyses were also reported.

The details of dependent and independent variables are shown in Table 2:

All statistical analyses were conducted using STATA version 10.0, and statistical significance was set at *P* < 0.05.

## 3 Results

### 3.1 Characteristics of participating outpatients

A total of 748,632 outpatient prescriptions were included in this study. The characteristics of participating patients were shown between the intervention and control groups both before and after the intervention (Table 3). The mean age of all participating patients was 37.51 years old (standard deviation = 24.46), with 38.64 (standard deviation = 23.98) years old for control group and 36.26 (standard deviation = 24.93) years old for intervention group, respectively; approximately half of these patients (50.21%) were male and over 94.34% were enrolled with health insurance.

**Table 3:**
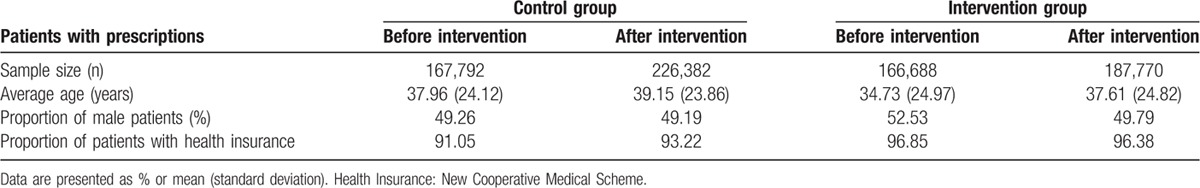
Characteristics of participating outpatients.

### 3.2 Characteristics of medicine use of outpatients

High rates of prescriptions requiring antibiotics or injections were noted in the intervention and control groups, but these rates decreased in both groups after the intervention. Moreover, the percentage of prescriptions requiring combined antibiotics increased in the control group and decreased in the intervention group. In addition, the average expenditure of prescriptions ranged from 35 to 51 Yuan (roughly USD$6.0–8.3) and slightly increased in both control and intervention groups after the intervention. Overall, the average expenditure of prescriptions was low for patients. Table 4 presents the details of the results.

**Table 4:**
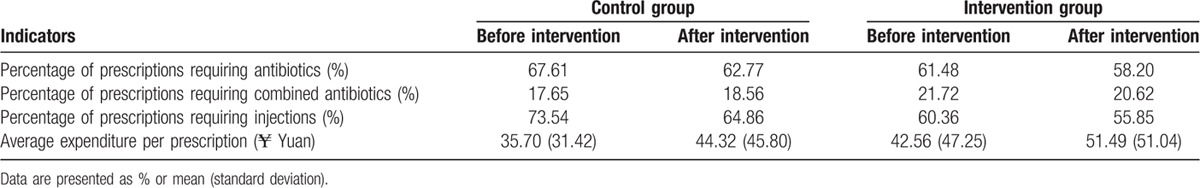
Characteristics of medicine use.

### 3.3 Evaluation of intervention effect

A regression analysis of the DID model was employed to evaluate the effect of PRHCP intervention. Adjusted estimates of PRHCP intervention were reported (Table 5), controlling the confounding from time, seasonality, and patient characteristics (age, sex, and enrolment with the NCMS). The PRHCP intervention led to a slight reduction in the use of combined antibiotics (odds ratio [OR] = 0.870, *P* < 0.001, 95%CI: 0.850–0.890) and slowed down the average expenditure increase of the patients (coefficient = −0.051, *P* < 0.001, 95%CI: −0.057 to −0.045). PRHCP intervention slightly increased the prescriptions requiring antibiotics (OR = 1.089, *P* < 0.001, 95%CI: 1.067–1.110) or injections (OR = 1. 258, *P* < 0.001, 95%CI: 1.234–1.283).

**Table 5:**
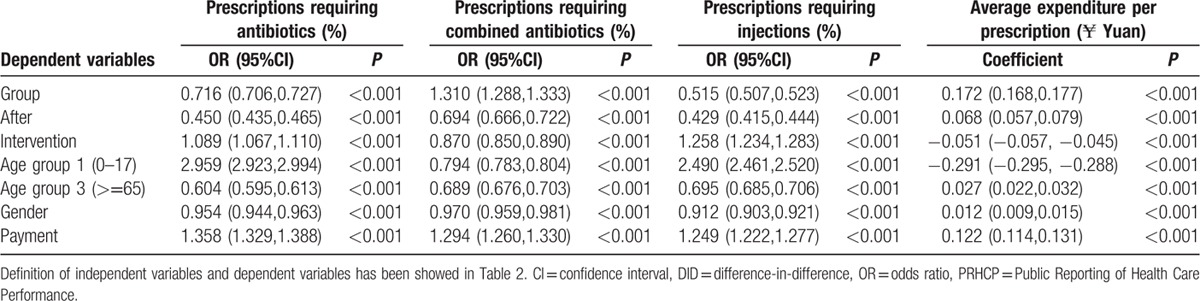
Evaluation of PRHCP intervention of all data by DID model regression analysis.

### 3.4 Evaluation of intervention effect in subgroups

To identify what kind of people would benefit from PRHCP intervention, 12 SGs (SG1–12) were classified according to gender, age, and health insurance ownership. A regression analysis of the DID model was applied to evaluate the PRHCP intervention effects in each SG, and time effect and seasonality were included for adjustment. All SGs have sufficient participant for DID regression model. Table 6 presents the details of the results.

**Table 6:**
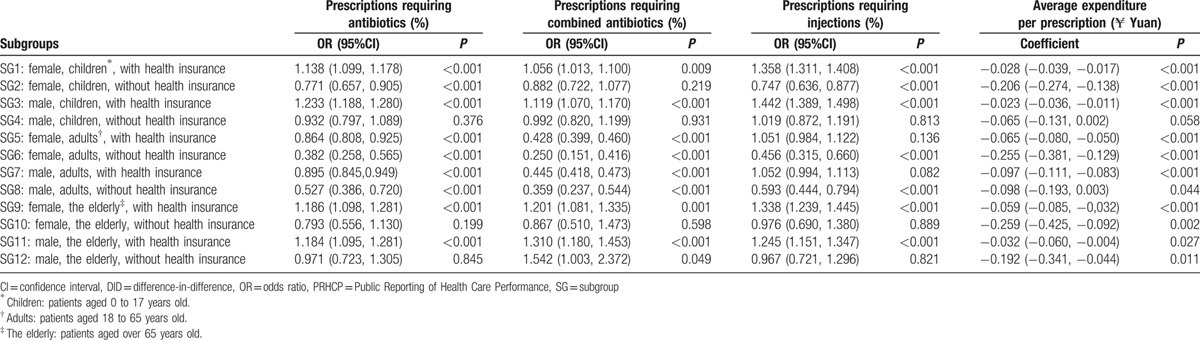
Evaluation of PRHCP intervention of subgroups’ data from DID model regression analysis.

PRHCP intervention influenced the use of antibiotics in most SGs except SG4, SG10, and SG12. However, the effect was mixed. The results of 18 to 64 years old patients (SG5–8) showed that PRHCP intervention decreased the probability of prescriptions requiring antibiotics (OR < 1). By contrast, PRHCP intervention only increased the antibiotic usage on elderly and minor patients who had health insurance (SG1, 3, 9, 11).

Similar effect was also found for combined antibiotic prescriptions. The difference between antibiotic prescriptions and combined antibiotic prescriptions is that PRHCP showed stronger effect decreased combined antibiotic usage in 18 to 64 years old patients, with OR < 0.45 (SG5–8).

A total of 7 SGs showed PRHCP intervention influenced injection usage. The increase injection use was reported in elderly and minor patients with health insurance (SG1, 3, 9, 11). Although the decrease injection use was found in 18 to 64 year old patients without health insurance (SG6, SG8) and female minor patient without health insurance (SG2). PRHCP reduced average expenditure of most SGs except for SG4.

Overall, PRHCP increased antibiotic and injection use in all elderly and minor patients with health insurance (SG1, 3, 9, 11), while this effect was inversed in patients aged 18 to 64 years old.

## 4 Discussions

### 4.1 Patient factors influenced the effect of PRHCP intervention

This study, using a randomized matched-pair trial design in real-world, demonstrated that PRHCP intervention can influence the prescribing pattern of physicians. However, the effect of PRHCP intervention varied according to patients characteristics. When information of antibiotics and injection prescribing was available, elderly and minor patients with health insurance preferred to use more antibiotics and injections.

One important explanation for this phenomenon was that patients’ deep-rooted misconception toward antibiotics and injections. The persistent high prevalence of antibiotic and injections overuse has lasted for many decades^[35–37]^ and reflected both inertia in physician practice and continued demand from patients.^[3]^ Previous studies showed that Chinese health literacy is low^[38]^ and antibiotics and injections are considered as stronger, faster, and high-quality medicine for treatment.^[37]^ Furthermore, the misunderstanding of patients and prevalence of overuse antibiotics and injection imposed pressure on physicians to prescribe more, not fewer, antibiotics and injections.^[2]^ Without sufficient education, patients would choose high antibiotic or injection prescribing rate physicians as their priority selection.

By contrast, it was interesting that PRHCP reduced antibiotic and injection usage in patients aged 18 to 65 years old. Since there was no decrease in minor patient who were always taken to primary care institutions by their parents, adults patients seemed prefer antibiotics and injections only for their children. There is a conjectural reason for these results. After dissemination of the potential risk of overuse of antibiotics and injections for many years,^[39]^ the conception of young adults slightly changed. Nevertheless, these medicines were still considered as integral of high-quality care inveterately and were required to children by their patients.^[2]^

Moreover, physicians’ clinical practice varied according to the age of patients,^[27]^ which would contribute to this result. Similar result has been showed from a multicenter observational study about neonatal intensive care unit in 2011. Although there is no clinical indicator for supplemental parenteral nutrition, most clinicians still prescribed the treatment because of the anxiety from themselves and pressure from children's parents.^[40]^ This situation still plays an important role under current clinical practice.

Besides, health insurance also played a vital role in the increasing use of antibiotics and injection. Studies carried out in the late 1990s in China had provided evidence that medical insurance encouraged prescription of more items and antibiotics^[37,41,42]^ and a review synthesizing research showed that patients become less willing to seek care when plans increase copayments in the USA.^[43]^

Meanwhile, Chinese governments are intended to achieve universal health insurance coverage, and 3 types of health insurance, aiming at different population, have been established. The official data showed that over 95% people had enrolled in health insurance by the end of 2014.^[44]^ Considering the prevalence of health insurance coverage and patients attitude toward antibiotics or injections,^[3,37]^ PRHCP seemed to be harmful and might contribute to the irrational use of antibiotics and injections. Patient education, aiming at radically changing attitudes, should be taken to reverse the potential harmful trend.

PRHCP intervention reduced the average expenditure of patients in all SGs, though some of the results were not statistically significant (Table 6). Unlike the indicator of quality of prescriptions, the expense indicator was sensible to all patients.^[3]^ Since PRHCP showed entirely different effect for different patients, treating patients as a whole for public reporting may not be wise. PRHCP intervention must be designed for different patients.^[45]^

### 4.2 Indicators of PRHCP may influence the effect of intervention

The objective of PRHCP intervention in this study was to address the irrational use of antibiotics and injection. Thus, physicians’ prescribing performance and physicians prescribing behaviors were selected as reported information and outcome measurement, respectively. These 2 parts were directly related to ensure effective PRHCP intervention. However, studies about the New York State Cardiac Surgery Reporting System (NYSCSRS)^[19,46–48]^ showed that the effect of PRHCP varied when using different outcome indicators for evaluation. NYSCSRS, publicly reporting adjusted mortality rates, reduced patients’ death in all New York hospitals,^[19]^ but failed to promote market share redistribution,^[46–48]^ which means that NYSCSRS promoted care quality improvement, but not medical-behavior improvement. The studies on NYSCSRS demonstrated the importance of selecting outcome indicators of PRHCP. For policy makers and health administrators, the goal of PRHCP and reported information should be directly relevant.^[18,49]^

### 4.3 Design and implementation issues may affect the effect of PRHCP

Design and implementation issues could increase the effect of PRCHP on effectiveness. The PRHCP intervention that was designed and implemented by our team, a 3rd party, is different from other studies.^[17,18,23,50]^ Associations or colleges are more professional compared with the government. Thus, patients and physicians preferred to accept the information released by a 3rd party that was fair and credible and the buy-ins of patients and physicians in PRHCP intervention is essential for its success.^[3,51,52]^

In addition, it was rare to find a study using a randomized control trial design to explore PRHCP effect within a complex real-world environment and most existing research applied before–after or retrospective design.^[18,53,54]^ Evidence from randomized control trials was considered as the most robust and reliable^[3]^ and the different design methods may influence the results.

Knowledge on medication use was included to easily understand the released information, and a publicity strategy was considered for the wide dissemination of information during implementation. These efforts may contribute to the final effect of PRHCP intervention.^[14,18,23,31,32]^

Overall, many design and implementation details of PRHCP intervention was reported in this study, which may contribute to a future study design. To make PRHCP work as expected, impact, reliance, and acceptability of reporting information must be considered when choosing which information should be reported. These 3 aspects decide whether the information would be used or not. Dissemination is also an important part for PRHCP since most patients would not come to see the information specially when they come to healthcare institutions to see a physician. Furthermore, further research should be conducted on the effect of report design and implementation.^[18]^

## 5 Limitation

First, this study sites were primary care institutions. Therefore, the conclusions drawn from this research must be carefully generalized to other types of healthcare institutions. Second, this study explored the potential effectiveness of PRHCP intervention, but we did not attempt to empirically examine the causal pathways through which PRHCP influences the quality of care. The mechanism of PRHCP intervention is needed for further studies. Third, it may need some time for PRHCP intervention fully working as expected and early evaluation would overestimate the actual effect of PRHCP. Forth, though tried to control the confounding from investigators, we did not included characteristics of investigators in DID model, which could had potential bias for the results.

## 6 Conclusions

This study demonstrated that PRHCP intervention can influence the prescribing pattern of physicians, but the effect varies among patients with different characteristics. Elderly and minor patients with health insurance preferred to receive more antibiotics and injection after this information released, while this effect was inversed in 18 to 64 year old patients. Age and health insurance are 2 important factors that determine whether PRHCP will work as expected. Moreover, the design, implementation, and indicators of PRHCP may also influence the impact of the intervention. The results suggest that PRHCP intervention must be designed for different patients, and publicly reported information should be chosen based on the goals and also be directly relevant to the goals. Patient education, aiming at radically changing attitudes toward antibiotics and injections, should be taken to promote the effectiveness of public reporting in China. Further research is needed on the effect of report design and implementation.

## Acknowledgments

The authors thank the managers of the participating institutions. The authors also thank the local governments for the support.

## Supplementary Material

Supplemental Digital Content
