# Supplementary material for: Does public reporting influence antibiotic and injection prescribing to all patients? A cluster-randomized matched-pair trial in china
Source: Medicine (Baltimore). 2016 Jul 1;95(26):e3965. doi: 10.1097/MD.0000000000003965 (PMC4937909; doi:10.1097/MD.0000000000003965)
Supplement: Supplemental Digital Content [file medi-95-e3965-s001.docx]

**Table A. Outcomes and rank of quality indicators of providers of H primary care institution (2014. 3)**

There is an example of public release information, which had been translated to English. Table A shows an example of public release information in H primary care institution for physicians in March 2014.

| **Table A.** **Outcomes and rank of quality indicators of providers of H primary care institution (2014.3)** | | | | | | | |
| --- | --- | --- | --- | --- | --- | --- | --- |
| Departments | Name | Percentage of prescriptions requiring antibiotics (%) | Rank | Percentage of prescriptions requiring injection (%) | Rank | Average expenditure of patients(￥ yuan) | rank |
| Obstetrics and Gynecology | A1 |  |  |  |  |  |  |
|  | A2 |  |  |  |  |  |  |
|  | A3 |  |  |  |  |  |  |
| Ophthalmology and Otorhinolaryngology | B1 |  |  |  |  |  |  |
|  | B2 |  |  |  |  |  |  |
| Internal Medicine department | C1 |  |  |  |  |  |  |
|  | C2 |  |  |  |  |  |  |
|  | C3 |  |  |  |  |  |  |
| surgical department | D1 |  |  |  |  |  |  |
|  | D2 |  |  |  |  |  |  |
|  | D3 |  |  |  |  |  |  |

**Table B. Outcomes and rank of quality indicators of providers of H primary care institution (2014. 3)**

There is an example of public release information, which had been translated to English. Table B shows an example of public release information in H primary care institution for patients in March 2014.

| **Table B. Outcome and rank of quality indicators of providers of H primary care institution (2014.3)** | | | | | | | |
| --- | --- | --- | --- | --- | --- | --- | --- |
| Departments | Name | Percentage of prescriptions requiring antibiotics (%) | Rank | Percentage of prescriptions requiring injection (%) | Rank | Average expenditure of patients(￥ yuan) | rank |
| Obstetrics and Gynecology | A1 |  |  |  |  |  |  |
|  | A2 |  |  |  |  |  |  |
|  | A3 |  |  |  |  |  |  |
| Ophthalmology and Otorhinolaryngology | B1 |  |  |  |  |  |  |
|  | B2 |  |  |  |  |  |  |
| Internal Medicine department | C1 |  |  |  |  |  |  |
|  | C2 |  |  |  |  |  |  |
|  | C3 |  |  |  |  |  |  |
| surgical department | D1 |  |  |  |  |  |  |
|  | D2 |  |  |  |  |  |  |
|  | D3 |  |  |  |  |  |  |

Note:

1. Prescribing indicators were calculated using data extracted from the electronic health information system on a monthly basis. All the results were calculated by school of medicine and health management, Tongji Medical College of Huazhong University of Science and Technology.

2. Irrational drug prescribing contributes to bacterial resistance and adverse reaction. Adverse reaction and hospital-acquired infection of antibiotic-resistant bacteria has the capacity to increase medical expenditure, length of hospital stay, and ultimately patient mortality.

| **Table C. Outcomes and rank of quality indicators of institutions of 10 primary care institutions (2014. 3)**  There is an example of public release information, which had been translated to English. Table C shows an example of public release information of primary care institutions for patients and physicians in March 2014.  **Table C. Outcome and rank of quality indicators of institutions of 10 primary care institution (2014.3)** | | | | | | |
| --- | --- | --- | --- | --- | --- | --- |
| institutions | Percentage of prescriptions requiring antibiotics (%) | Rank | Percentage of prescriptions requiring injection (%) | Rank | Average expenditure of patients(￥ yuan) | rank |
| A primary care institution |  |  |  |  |  |  |
| B primary care institution |  |  |  |  |  |  |
| C primary care institution |  |  |  |  |  |  |
| D primary care institution |  |  |  |  |  |  |
| E primary care institution |  |  |  |  |  |  |
| F primary care institution |  |  |  |  |  |  |
| G primary care institution |  |  |  |  |  |  |
| H primary care institution |  |  |  |  |  |  |
| I primary care institution |  |  |  |  |  |  |
| J primary care institution |  |  |  |  |  |  |

Note:

1. Prescribing indicators were calculated using data extracted from the electronic health information system on a monthly basis. All the results were calculated by school of medicine and health management, Tongji Medical College of Huazhong University of Science and Technology.

2. Irrational drug prescribing contributes to bacterial resistance and adverse reaction. Adverse reaction and hospital-acquired infection of antibiotic-resistant bacteria has the capacity to increase medical expenditure, length of hospital stay, and ultimately patient mortality.
